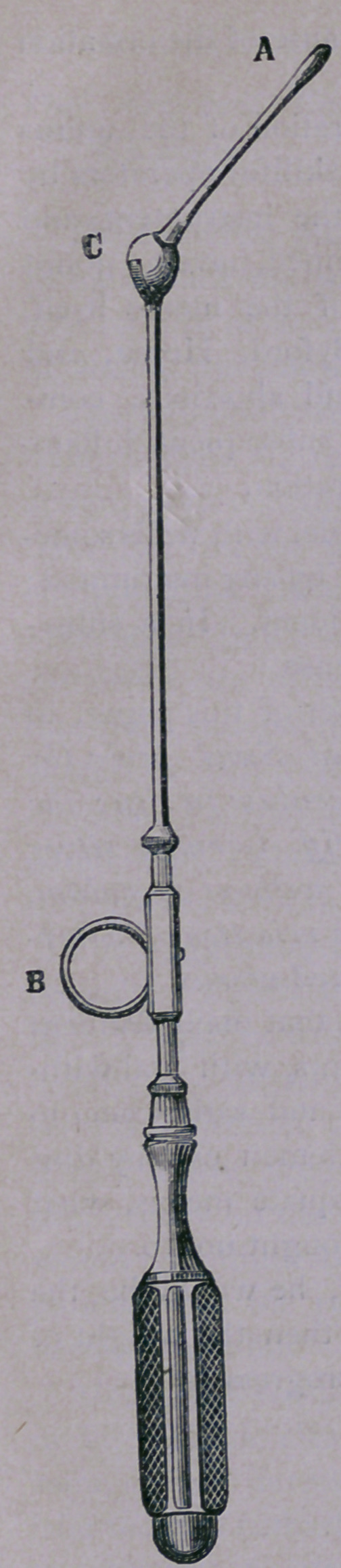# On a Few Instruments for the Practical Treatment of Uterine Disease

**Published:** 1871-03

**Authors:** Philip Adolphus

**Affiliations:** 169 Dearborn Street; Chicago


ARTICLE III.—*On a few Instruments for the Practical Treatment of Uterine Disease.*—By PHILIP ADOLPHUS, M.D., Chicago.

(Continued from February No.)

SIMPSON'S SOUND.
SIMS' UTERINE PROBE.
SIMS' UTERINE ELEVATOR.

When a man has become accustomed to the use of one instrument, he feels awkward when a new one is placed in his hands, even though it may be vastly superior to the old, and is therefore loth to adopt it. A valuable discovery or invention often requires a long period to secure its acceptance by even the most enterprising of our profession. It is not surprising, then, that the mass should be slow to adopt improvements. Thorough acquaintance is necessary to appreciation.

The Uterine Sound and the Uterine Probe are illustrations in point. "Reviving simultaneously an old method of diagnosis which had been described in 1828 by Lair; Simpson, Hugier and Kiwisch, in 1848, claimed its discovery" (T. G. Thomas, Diseases of Women, 1869). We are indebted to Simpson for its application to uterine pathology. His teachings regarding the mode of using the probe are to-day just as satisfactory to the mass of the profession, and are practiced and expounded by its leaders in their text books, from Bennet, 1852, to Hewit, 1869, with as much force and unction as ever; despite its modification in consistence, shape, and manner of introduction, by Sims in 1862, and the reduction of its size by Emmet still later. Thomas alone deserves the credit of having done that justice to his contemporary that all knew was his due.

Every one is acquainted with Simpson's instrument. It is a strong metallic sound, of German silver, 10 inches in length, and of the size of a No. 3 catheter, terminated by a slight bulb on one end, and by a flat handle at the other; at 2½ inches from the extremity of the instrument, there is placed a slight knob.

The mode of introduction of Simpson's sound is as follows: The patient being placed on her back, the forefinger of the right (or left) hand is introduced into the vagina, and its extremity brought in contact with the cervix uteri, so as to act as a guide to the point of the instrument. This, previously oiled, is held in the left (right) hand, and its point slipped along the palmar surface of the finger in the vagina, and directed by it into the uterine orifice. By depressing the handle, the point will slip along the cervix. If the proper curve have been given the instrument, its passage from the cervix into the cavity of the uterus to the fundus will then be effected.

The description of its mode of introduction is plain, but its performance is accomplished with difficulty, when deviations of the normal axis of the canal are present, and these are the cases which we are called on to examine by the sound. The difficulties of introduction in versions, increase largely in flexions, or when the cavity is distorted or partly obliterated by neoplasms, the tortuosity of the canal assuming hundreds of phases, each more trying and difficult to the operator. Care and gentleness, much patience, considerable dexterity, and great experience, are necessary on the part of the physician, and even then in many cases, irritations more or less serious, nausea, faintness, injury to mucous membrane, and violent pain, are inflicted upon the patient, so that Tilt advises an injection of 20 or 30 drops of laudanum after the introduction of the sound. Scanzoni considers it by no means a harmless instrument. Bequerel (as quoted by Thomas) dreaded seeing it popularized in general practice. Nonat says that it should be only inserted with great caution, in cases where the necessity of its use is clearly shown.

It is not alone the abnormal disposition of the uterine canal which create these difficulties; the instrument is too large, too straight, by many used in the same fixed curvature, thus producing great pain. Sims acknowledges "that he had often the greatest difficulty with the German silver sound," and states that "he had seen a score of cases in consultation where physicians assured him it was utterly impossible to pass the sound, and that he had seen such excessive pain thus inflicted, that the patient could hardly be persuaded to allow a repetition of the process."*

But thus deterred by the difficulty of its introduction, the physician who rejects its use, neglects to avail himself of a useful means of diagnosis, and deduces incorrect conclusions from insufficient data of information.

It may truly be considered an epoch in Gynecology, when Sims introduced his speculum. Then for the first time could Simpson's sound be handled with safety and ease, the surgeon's eye guiding his hand; the *sound* thus used of necessity became a *probe.* It traced out the malpositions of the womb as painlessly as the surgeon's probe traces a sinus; it did more; it made the introduction of all instruments into the uterus certain, easy, painless, and the consequent treatment satisfactory; in short, after being modified by Emmet, it became, in competent hands, a delicate, nervous, insinuating, obedient finger.

Thus the probe—small, smooth, pliable—is Simpson's sound modified by Sims. He supplanted the labors of his illustrious predecessor, and has thus been enabled to cultivate the master's labors, multiplying the good received a hundred fold, to the glory of Simpson's memory, the advantage of the profession, and the benefit of our patients.

* Clinical Notes on Uterine Surgery, by M. Sims, fol. 20, 22, 23.

*Sims' Probe, modified by Emmet.*

The following teachings regarding the uterine sound, which emanated from the comprehensive mind of that illustrious man 28 years ago, are as true to-day as they were then. These teachings are contained in the text books of the day, and are easily demonstrated in practice by Nott's speculum and Sims' probe. They are as follows:

The probe detects—

(*a*) The alterations in the direction of the uterine canal.

(*b*) The connection of the uterus with pelvic growths.

(*c*) The existence of diseased states of the cavity, fundus, and walls of the organ.

(*d*) The existence of growths within it, and its mobility.

In the shops of to-day can be found Simpson's sound; also a plain, large, non-graduated, smooth probe, it is Sims'; and the still smaller probe of Emmet, which is now used by Sims. It is the latter to which the writer refers whenever he speaks of Sims' probe.

This instrument is made of silver, or of annealed copper, silvered. It is eight inches in length, of the thickness of a medium sized copper wire, without marks or notches, terminating in a bulbous extremity, and is mounted in a light wooden handle three inches long.

Simpson's instrument is used as a *sound*, that of Sims' as a *probe*, to measure the depth of the uterus, and to show in what direction the fundus lies (Sims); the writer also employs it *as a guide*, in order to convey all kinds of instruments into the uterus with ease, certainty and safety. The mode of its introduction is as follows (after having examined the patient by *bi-manual palpation, which should always precede any examination of the uterus*, for the purpose, amongst other reasons, of ascertaining the direction of the womb): introduce Sims' or Nott's speculum; if the former, place her in the semi-prone position; if the latter, on her back; draw down and steady the cervix with a hook (Sims') or tiny vulsellum (Nott's) introduced into its anterior lip. (We will suppose, that owing to spasmodic contraction of the abdominal parietes, fatty deposit, painful condition of parts, or for any other reason, we have not been able to ascertain the direction of the womb in our first examination by bi-manual palpation). Take the probe in the right hand, and slowly, gently, patiently, with a light hand, use it precisely as a surgeon would probe a sinus. Bend the probe and introduce it in the position the womb ought to occupy if healthy; if it is arrested, insinuate it in a more bent position, for perhaps the womb is anteverted; if it is again arrested, introduce it acutely bent, with the point forward and the handle turned towards the sacrum, for perhaps it is anteflexed. If success does not follow, and the womb is known to be extremely anteflexed, take a couple of Sims' sponge-holders, arm them, place them into the anterior cul de sac and under the anteflexed fundus, and patiently coax it to raise itself; it will then be possible to introduce the probe. Or again, if the womb be retroverted, the point of the probe is to be directed towards the sacrum; if it meets with an obstacle, it is to be removed and re-introduced in a different curvature. It will be found that in many cases of flexions, that the probe for half an inch or an inch is introduced in the normal manner, when suddenly the womb is found to be anteflexed, retroflexed, or assumes a right or left latero-flexion, and the probe will have to be bent in the corresponding direction.

In acute flexions of the organ it is sometimes impossible to introduce the probe until the canal has been somewhat straightened by lifting up the fundus, either from the bladder or rectum, by means of small pieces of sponge, inserted in Sims' sponge-holder, and placed in Douglas' fossa, the rectum, or in the anterior portion of the vagina. Two of these sponge-holders persuading the womb, will slowly replace the organ sufficiently to allow of minute probing and applications even if it is bound down by adhesions.

If we cannot succeed in introducing the probe *without pain*, it is best to postpone the introduction to a future period, and apply glycerine suppositories if the parts are tender or inflamed.

As a general rule, the operator will succeed with more or less ease, *provided he exercises prudence and patience, and in difficult cases does not insist upon completing the diagnosis at the first examination.*

*The probe should not be introduced without a proper speculum in situ.*

*The bi-manual examination will always produce more pain* (if pain is felt at all) *than the probing of the womb, even in difficult cases.*

*The probing of the cavity of the womb as a means of confirming the diagnosis made by the touch should be considered in every case of uterine disease, an essential constituent of the examination of the patient, not merely as a means of diagnosis, but also of treatment* (vide Sims, Thomas). *The contra-indications to this imperative rule will, however, be stated anon.*

No one can diagnose diseases of the pelvis, and especially inflammations of the womb, with their accompanying complications, with justice to himself and his patients, unless he becomes an adept in the use of the uterine probe.

Knowing that the curve of the diseased womb varies exceedingly in different cases, the writer has derived great assistance in preceding the introduction of any instrument into the womb, by the probe, and has thus succeeded in rendering treatment *comparatively* painless in his hands. He, therefore, can recommend with confidence, to the profession, *the use of the probe as a guide* to every instrument to be introduced into the cervix, body or fundus of the womb. In order to elucidate the method adopted, he will illustrate it. Take, for example, a case of corporeal metritis and endometritis.

1st. Introduce probe for diagnosis; womb five inches, direction normal.

2d. Introduce probe, to be left in situ; insinuate gently into cervix Nott's cathether, for the purpose of cleaning the womb; if it can enter with ease, remove probe, pass in catheter and wash out cavity with warm-salt water; if not, while withdrawing probe, introduce Nott's dilator, and if dilatable, gently dilate cervix at once, gradually and slowly, telling the patient to inform you when it hurts her *much*, during dilatation.

3d. Introduce probe, followed by Nott's catheter; womb cleaned.

4th. Introduce probe, followed by Emmet's applicator, armed with a pledget of cotton saturated with glycerine, followed by a suppository of cotton wool saturated with glycerine, placed under cervix; both to be left in situ for twenty-four hours.

Finally, Introduce a rectal pill of Morphia and Belladonna.

This treatment is repeated daily, until cavity of womb is less tender, and cleaner. Of course in all cases proper constitutional treatment is given.

Then repeat Nos. 1 and 2; the catheter is followed by the probe, that by Pinkham's Intra-Uterine Scarificator; that by the probe, for the purpose of applying, by means of Emmet's applicator, an intra-uterine suppository of cotton charged with glycerine; another is placed in the vagina, and the patient arises from the chair, despite the constant probings, with less irritation than if it had not been used; because all the instruments have performed their office as painlessly as possible, no bungling having been tolerated; the probe previously introduced has permitted them to follow the identical channel just as painlessly, owing to previous dilation, if necessary. Many readers will doubt that this mode of using the probe can be as painless as the writer represents it, therefore he will hold himself in readiness to demonstrate this whenever called on to do so.

Let us recapitulate. The three fundamental requisites of efficient uterine diagnosis and treatment are, bi-manual palpation, a proper speculum (Sims' or Nott's, described in the February number of this journal), and the probe used as a probe and a guide.

The restoration of the retroverted uterus to its normal position by Simpson's sound, is fraught with difficulty, always with pain and hæmorrhage, sometimes with danger of perforation of the fundus. The retroversion is diagnosed by the introduction of the sound, the instrument is then turned for half a circle, the fundus is elevated towards the sacrum, during which operation the whole weight of the organ is supported on the point of the instrument.

It is much safer to reduce these flexions by means of position, the hand, armed probangs, etc. The sound should, according to Tilt, Sims, and Thomas, not be used for that purpose.

Sims' Uterine Elevator is a useful instrument, constructed on correct principles, and preferable to Simpson's sound.

* It is a sound; two inches from its uterine extremity is a hinge joint, on which is placed a disk. The instrument is 13 inches long, and consists of a handle (3 inches), a shaft (7 inches), and a uterine stem 2¼ inches, inserted into a disk which revolves at the end of the shaft. The disk is perforated by seven holes (the stem occupying the eighth). The shaft is a hollow cylinder containing a rod, which is retracted or pushed forward at will, so that its point may lodge in any one of the perforations in the disk, whereby the stem may be held firmly at any angle with the shaft.

It is used as follows: Introduce the probe for diagnosis, adjust the stem of the Elevator at the proper angle with the shaft, introduce it into the uterus, press the disk close up the os tincæ, push the mouth of the womb downwards and backwards in the direction which was, at the reception of the manœuvre, occupied by the fundus.

"This instrument is simply Simpson's sound with a joint or hinge two inches from its uterine extremity; but its *modus operandi* is very different. One elevates the uterus in a right line, the other in a circle to the right or left; one supports the weight of the organ on a ball at the os, the other principally on the point of the sound in the uterine cavity; one elevates the uterus by a power exerted on the cervix, the other by a like power on the fundus; one seldom produces any pain, the other often does." †

The contra-indications to the use of the sound and probe are, pregnancy, suspected pregnancy, *fatty degeneration of the uterus, accompanied by a state of excessive*

* Vide Op. Cit. and Am. Journal Med. Sciences, January, 1858.

† Sims' Op. Cit., fol 260.

*superinvolution;* and amenorhœa, until the cause of the cessation of the menses is ascertained.

In conclusion, many of the cases of perforation of the uterus reported to have happened in the practice of skillful operators by means of Simpson's sound, and for which the instrument and the mode of its introduction have been condemned, have no doubt been caused by the pathological condition of the uterus itself, which, according to Simpson, Scanzoni, Byford, Hewit, and especially Klob, after frequent pregnancies and abortions, complicated and followed by puerperal fever or subsequent inflammation, has become atrophied by fatty degeneration.

A uterus thus diseased, flaccid, softened, friable, with walls sometimes as thin as paper, is liable to be perforated by any instrument; and the utmost care will not prevent this accident. In a subsequent paper this subject will be treated in extenso.

Finally, it has been the object of the writer of this paper, to suggest the *general adoption of the modified Sims' probe (by Emmet); its mode of introduction with the speculum in situ; the rejection for purposes of diagnosis of the stiff German silver Simpson sound, and its mode of introduction without speculum, as now taught in the text books; and the entire rejection of Simpson's sound as a redressor in cases of retroverted uterus.*

It must, however, be admitted by the writer, that many eminent men pass Simpson's sound and other instruments, with and without specula in situ, with safety to the patient and satisfaction to themselves. This only proves that these gentlemen can by dint of practical manipulation perform, with inadequate means, what the general practitioner will not attempt, and ought not to.

If the illustrious Simpson were yet with us, he would be the first to adopt the latest improvements; let not then the weight of his great reputation stand in the way of real progress.

169 Dearborn Street.

## Figures and Tables

**Figure f1:**
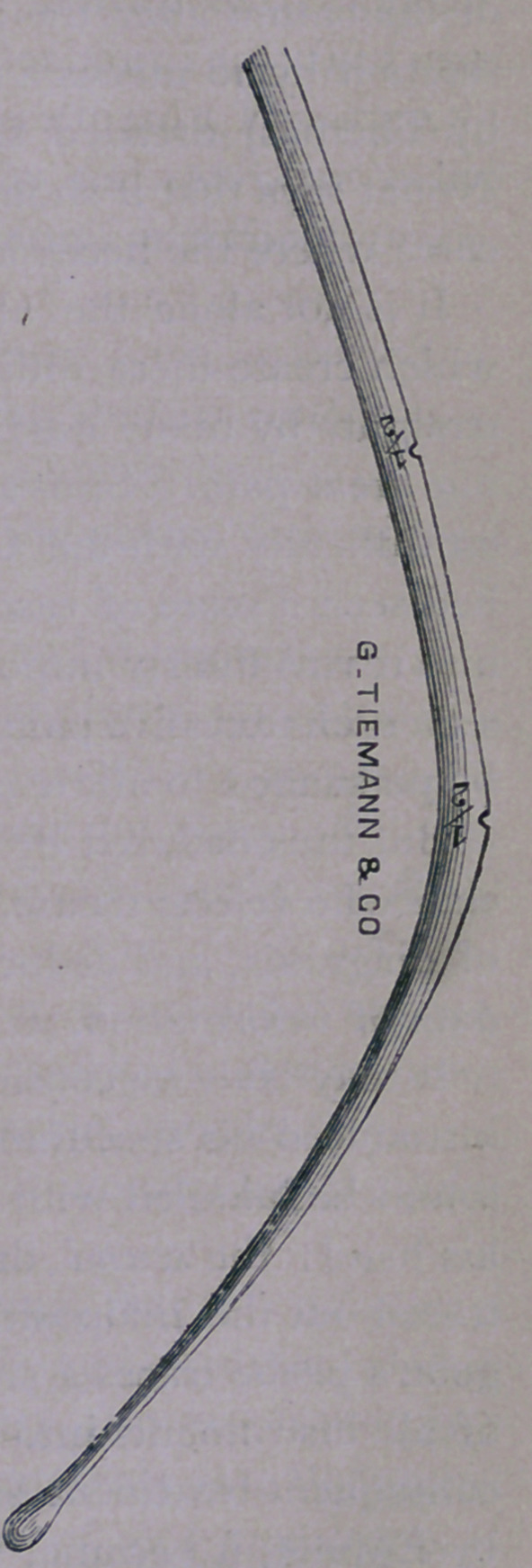


**Figure f2:**
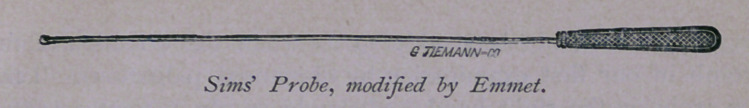


**Figure f3:**